# Field-Dependent Stiffness of a Soft Structure Fabricated from Magnetic-Responsive Materials: Magnetorheological Elastomer and Fluid

**DOI:** 10.3390/ma13040953

**Published:** 2020-02-20

**Authors:** Byung-Keun Song, Ji-Young Yoon, Seong-Woo Hong, Seung-Bok Choi

**Affiliations:** 1 Department of Mechanical Engineering, Incheon National University, Incheon 22012, Korea; 2 Department of Mechanical Engineering, Inha University, Incheon 22212, Korea; ji_young62@naver.com (J.-Y.Y.); ghdtjddn3@gmail.com (S.-W.H.)

**Keywords:** soft structure, magnetorheological elastomer, skin layer, magnetorheological fluid, stiffness change, tensile test, rectangular channel, deformation

## Abstract

A very flexible structure with a tunable stiffness controlled by an external magnetic stimulus is presented. The proposed structure is fabricated using two magnetic-responsive materials, namely a magnetorheological elastomer (MRE) as a skin layer and a magnetorheological fluid (MRF) as a core to fill the void channels of the skin layer. After briefly describing the field-dependent material characteristics of the MRE and MRF, the fabrication procedures of the structure are provided in detail. The MRE skin layer is produced using a precise mold with rectangular void channels to hold the MRF. Two samples are produced, namely with and without MRF, to evaluate the stiffness change attributed to the MRF. A magnetic field is generated using two permanent magnets attached to a specialized jig in a universal tensile machine. The force-displacement relationship of the two samples are measured as a function of magnetic flux density. Stiffness change is analyzed at two different regions, namely a small and large deformation region. The sample with MRF exhibits much higher stiffness increases in the small deformation region than the sample without MRF. Furthermore, the stiffness of the sample with MRF also increases in the large deformation region, while the stiffness of the sample without MRF remains constant. The inherent and advantageous characteristics of the proposed structure are demonstrated through two conceptual applications, namely a haptic rollable keyboard and a smart braille watch.

## 1. Introduction

**Figure 1:**
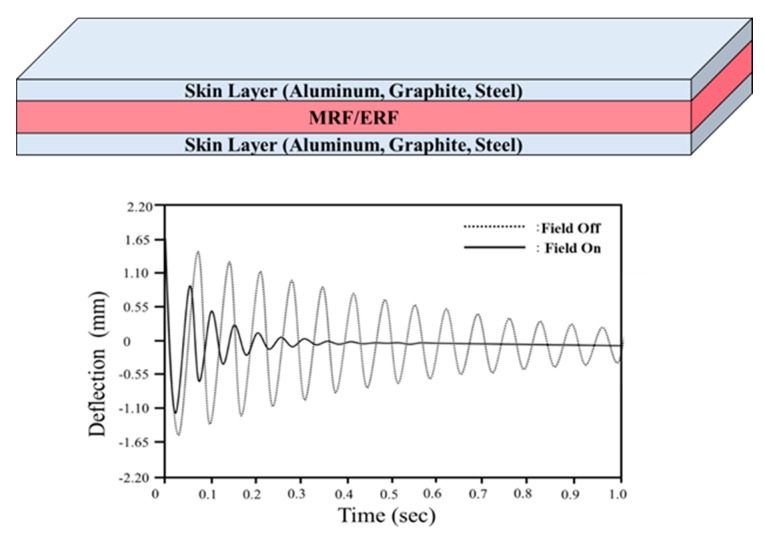
A typical smart structure using controllable magnetorheological fluid (MRF)/ electrorheological fluids (ERF) for vibration control.

Smart fluids, including electrorheological fluids (ERFs) and magnetorheological fluids (MRFs), can be effectively applied in a number of scenarios, including as an automotive damper and for vibration control in flexible structures. ERF consists of a carrier liquid with fine nonconducting particles, and its apparent viscosity depends heavily on an electric field applied to the fluid domain. Thus, ERF is often utilized as a core layer in sandwich structures (e.g., beams, shells or plates) to improve damping properties or to modify the modal characteristics of a structure [1,2,3]. However, a very high electric field of 2 kV/mm is required for optimal performance. MRFs have become a popular alternative in low current and low voltage applications. MRF consists of a carrier liquid with iron particles and has similar rheological characteristics to ERF as a function of the magnetic field. As MRF produces a much higher yield force than ERF, it has been used in a wide variety of applications, including the control of unwanted vibrations in flexible structures. MRF may be placed as a damping core (i.e., constraint layer) between two outer structures (i.e., skin layers) and is responsive to a magnetic field [4,5,6]. ERF and MRF are often only used for vibration control of flexible beams and plates because the outer components of smart structures are usually insufficiently soft or flexible. These structures cannot be used for stretchable or wearable devices due to their rigidity. The configuration of a conventional smart structure (i.e., composite) is shown in Figure 1, where the natural frequencies of the ERF or MRF core can be tuned based on the intensity of external stimuli. This tunable property is advantageous for the effective control of unwanted vibrations and is possible by avoiding the resonance problem with a specific magnetic field intensity. The transient vibration decreases to zero almost immediately when a magnetic field is applied to an MRF core domain.

Some applications, for example bio-health patches and wearable haptic gloves, require a soft outer skin layer. A magnetorheological elastomer (MRE) is a skin (i.e., matrix) comprising of rubber embedded in magnetic-responsive iron particles. The rubber component contributes sufficient softness to allow for the manufacturing of a wide range of shapes and curvatures. Previous studies have demonstrated the effectiveness of MRE-based structures [7,8]. Unlike an MRF, an MRE consists of a solid matrix that restricts the movement of the field-dependent iron particles. Research has focused on the use of various materials as alternatives to MREs for application in soft structures. Shan et al. [9] evaluated the stress–strain behaviors in a tunable composite using polydimethylsiloxane elastomer with a low melting point metal solder. The Young’s modulus of the soft composite was tuned by four orders of the magnitude with electronic activation. A smart composite for use in soft robots was recently introduced, where the application involved discretization with localized geometric patterns [10]. The stiffness controllability of the soft composite was evaluated by implementing shape memory alloy (SMA) at different temperatures. Accurate control of the stiffness or compliance of a soft composite is also crucial in the development of soft robotics [11], and control strategies such as open-loop or closed-loop control algorithms have been implemented to achieve high performance [12]. Reversible rigidity control has been demonstrated in laminar composites that contain a shape memory polymer [13] and the phase-changing metal alloy was effective in reversibly tuning the stiffness of the elastomer composites [14].

**Figure 2:**
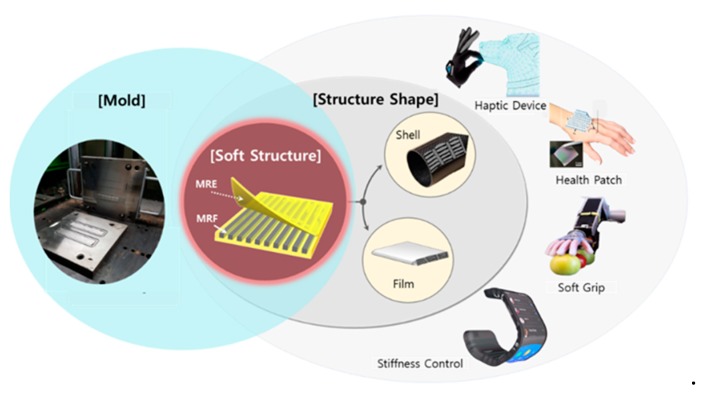
Applications of the proposed soft structures produced using two different magnetic-responsive materials.

The main technical contribution of this work is to fabricate a new soft structure whose stiffness can be tuned by external magnetic field. The field-dependent stiffness of the structure was evaluated experimentally. The structure consisted of two magnetic-responsive materials, namely MRE as a skin layer and MRF as a core to fill void channels in the skin layer. The MRE skin layer contained 30 wt.% of carbonyl iron particles (CIP) and was produced via a multiple step process, including molding and curing. The mold was made from machine structure carbon steel (S55C) and had excellent wear resistance. The mold had rectangular void channels into which MRF was injected, followed by bonding between the upper and lower skin layers. Two samples with identical geometry and dimensions were produced with and without injecting MRF into the channels. Magnetic analysis with permanent magnets was conducted to ensure that the magnetic field was well-distributed. The samples were fixed to a specialized jig in a universal tensile machine and the tensile force was detected by the load cell at the top, while the tensile length was determined using an embedded encoder attached to the motor. After several trial tests considering data collection, noise and breaking possibility of the samples, the tensile speed was fixed at 500 mm/min. The force-displacement curves of two samples were used to establish the stiffness change as a function of the magnetic flux density. The sample without MRF exhibited an increase in stiffness of 72.8% in a small deformation region when a 0.3 T magnetic field was applied, while the sample filled with MRF exhibited a 156.6% increase under the same conditions. The stiffness of the sample with MRF increased across a large deformation region, while the stiffness of the sample without MRF did not vary with the application of a magnetic field. Unlike the conventional smart structures shown in Figure 1, the proposed soft structure can be applied to a wider variety of fields. Examples of the proposed soft structure applications included wearable bio-patches, stiffness controllable gloves for use in virtual work or haptic devices, nerve stimulation treatment devices and localized variable stiffness control devices as shown in Figure 2. This work presents the preliminary results that demonstrate the proof of concept regarding the stiffness tuning capability of the soft structures as a function of magnetic field intensity. However, two conceptual application examples have been presented to attract further research interest in science and engineering, specifically a rollable keyboard with a haptic function and a smart braille watch for the visually impaired.

## 2. Fabrication of Soft Structures

### 2.1. Characteristics of MRE

**Figure 3:**
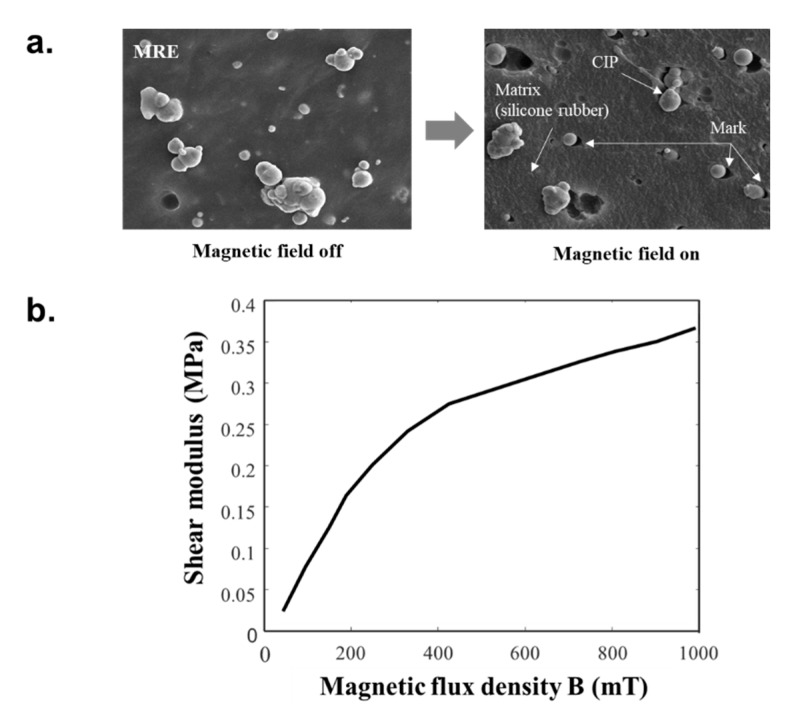
Material characterization of magnetorheological elastomer (MRE) presented as a (**a**) SEM image and (**b**) shear modulus vs. magnetic flux density plot.

An MRE is a composite material that reacts to a magnetic field. It has an elastic solid state in the absence of the magnetic field, as opposed to MRF which is fluid. However, MRE has been used in similar application fields to MRF, including the use as a vibration isolator. The magnetically sensitive particles in MRE were bound within a silicone rubber matrix and an additive as shown in Figure 3a. The scanning electron microscopy (SEM) images clearly display that carbonyl iron particles (CIP) shifted in the magnetic direction, similar to the effects of an inter-particle attraction force. When the magnetic field was applied, the carbon particles within the MRF provided a damping force by rearranging, while the magnetic-responsive particles in the MRE modulated the shear modulus of the material as shown in Figure 3b and provided a damping force via a magnetically engaging bond [15,16]. The production process of an MRE can be used to impart material properties in a specific single axis pattern by applying a magnetic field in a specific direction, which is referred to as an isotropic MRE. If this step is not conducted, an anisotropic MRE is produced. The magnetically responsive particles in an isotropic MRE are randomly and evenly distributed in the elastic structure, while the magnetically responsive particles in an anisotropic MRE have a straightened structure within the rubber matrix [17]. An isotropic MRE is used in the current study as it is easier to produce than an anisotropic MRE. After magnetizing in a strong magnetic field, carbonyl iron particles (CIP) were pressed in the magnetic direction, similar to the effects of an inter-particle attraction force.

### 2.2. Characteristics of MRF

**Figure 4:**
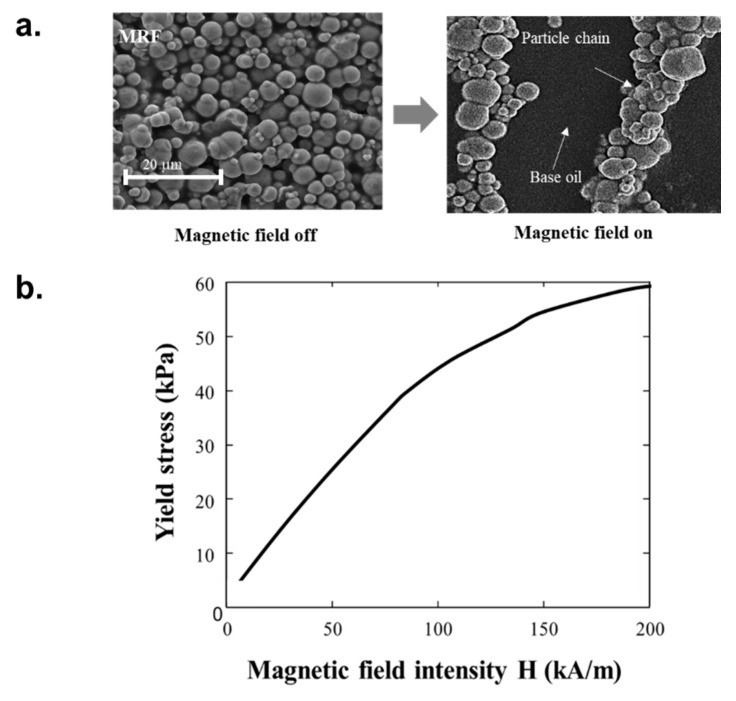
Material characterization of the MRF presented as an (**a**) SEM image and (**b**) yield stress vs. magnetic flux density plot.

MRF is a typical controllable intelligent fluid and is usually composed of a silicon oil-based fluid and ferrous particles that interact with a magnetic field. If an MRF is acted upon by the magnetic field, its yield stress can quickly change as the particles align to form chain-like structures. The main particles in MRFs are micro-sized. An MRF is generally treated as Bingham fluid, which is a type of non-Newtonian fluid [18,19]. Commercial MRF (MRF 140CG, Lord) was used to produce a specimen with uniform performance, and the SEM images of MRF (MRF 140CG, Lord) clearly show that the particles were randomly distributed without the magnetic field and have anisotropic arrangement with the magnetic field as shown in Figure 4a. The chain-like structures that form under a magnetic field cause the behavior of the MRF to shift from a Newtonian liquid to the Bingham model, where yield stress is generated within the fluid. This change in fluid properties occurs within milliseconds, which allows for use in dynamic applications that involve braking, damping or vibrating in a short period of time. The relationship between the magnetic field intensity and yield stress shown in Figure 4b indicate that the use of MRF in the proposed flexible structure can affect the dynamic stiffness change.

### 2.3. Sample Fabrication

**Figure 5:**
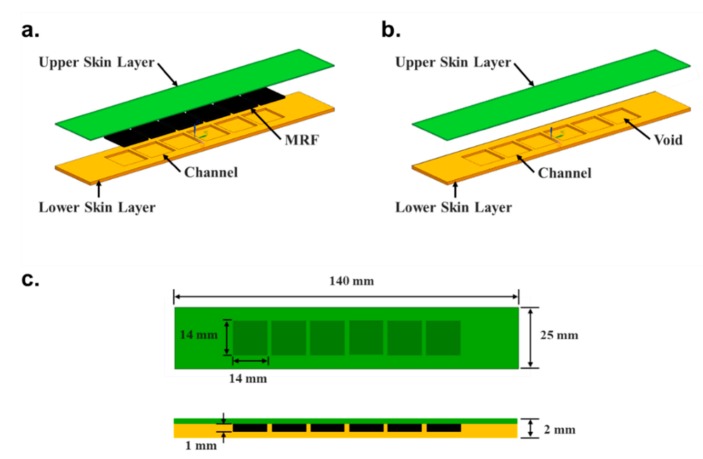
The two soft structure samples, namely (**a**) filled with MRF and (**b**) without MRF, and (**c**) their identical geometric dimensions.

Two samples were produced to demonstrate the effects of the MRF on the stiffness change in the soft structure. The configurations and geometric dimensions of the samples are given in Figure 5. The first sample consists of upper and lower skin layers with square channels containing MRF as shown in Figure 5a, while the second sample has the same upper and lower layers, but the channels contain no MRF as shown in Figure 5b. The samples were 140 mm long, 25 mm wide, 2 mm thick with six channels (refer to Figure 5c). The channels were 14 mm long, 14 mm wide and 1 mm deep with 2 mm spacing. The sample was gripped by the tester at either end (23 mm from edge).

**Figure 6:**
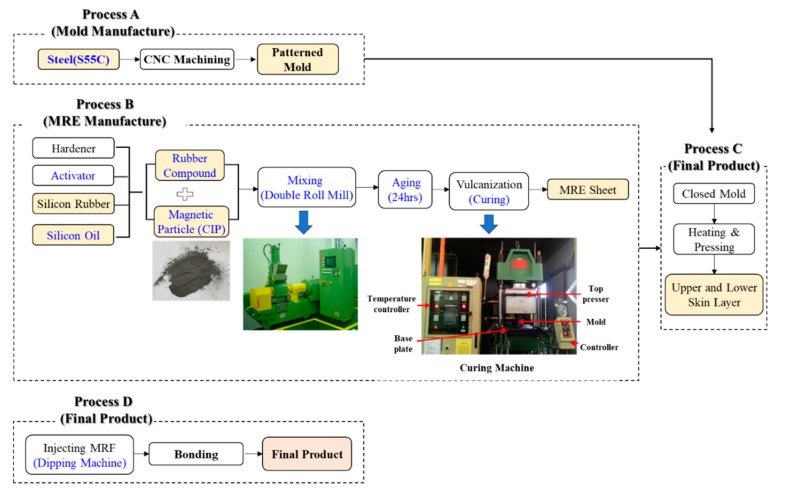
The manufacturing process for the soft structure samples.

**Figure 7:**
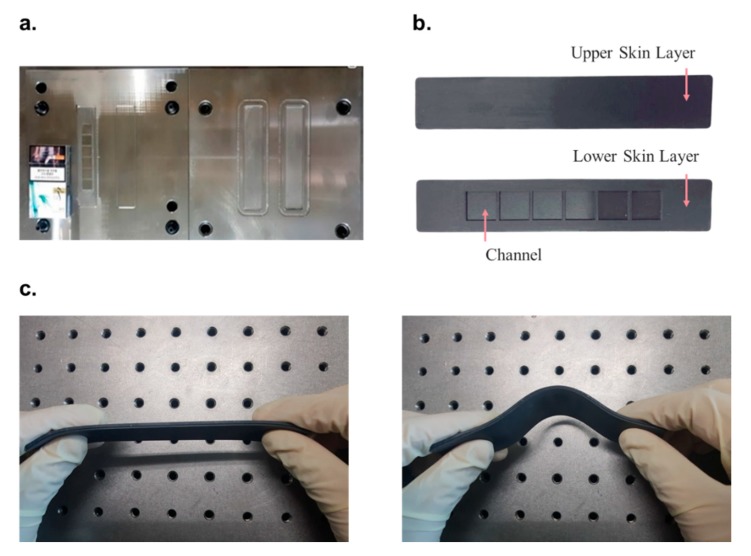
The sample and its components during production, namely the (**a**) mold, (**b**) skin layers and (**c**) assembled structure.

The production process was divided into four parts as shown in Figure 6. Process A was the mold manufacturing step, where molds for the upper and lower skin layers were produced using machine structure carbon steel (S55C) with excellent wear resistance (refer to Figure 7a). Process B involved manufacturing the MRE sheet, which was the material of the skin layer. A rubber compound containing hardener, activator, silicon rubber and silicone oil was mixed with 30 wt.% CIP in a double roll mill for 20 to 30 min to blend sufficiently and aged at room temperature for 24 h to stabilize the properties after the chemical reactions had ended. The compound was vulcanized to increase the elasticity and durability. Under 160 °C for 7 min, amount of sulfur added was adjusted to ensure a shore hardness of 30. The curing was performed without applying a separate magnetic field to the outside so that CIP was randomly distributed in the compound. A sheet of MRE was extruded. Process C involved forming the skin layer from the MRE sheet. The upper and lower mold plates were heated at 160 °C and the MRE sheet was pressed between the mold plates and removed (refer to Figure 7b). Process D involved the assembly of the components. An MRF-filled sample was formed by filling each channel of the lower skin layer with 1.96 cc of MRF (MRF-140CG, Lord) using a dipping machine. The sample without MRF was produced without this step. The upper and the lower skin layers were attached via silicon bonds to form a flexible structure as shown in Figure 7c.

## 3. Experimental Apparatus

**Figure 8:**
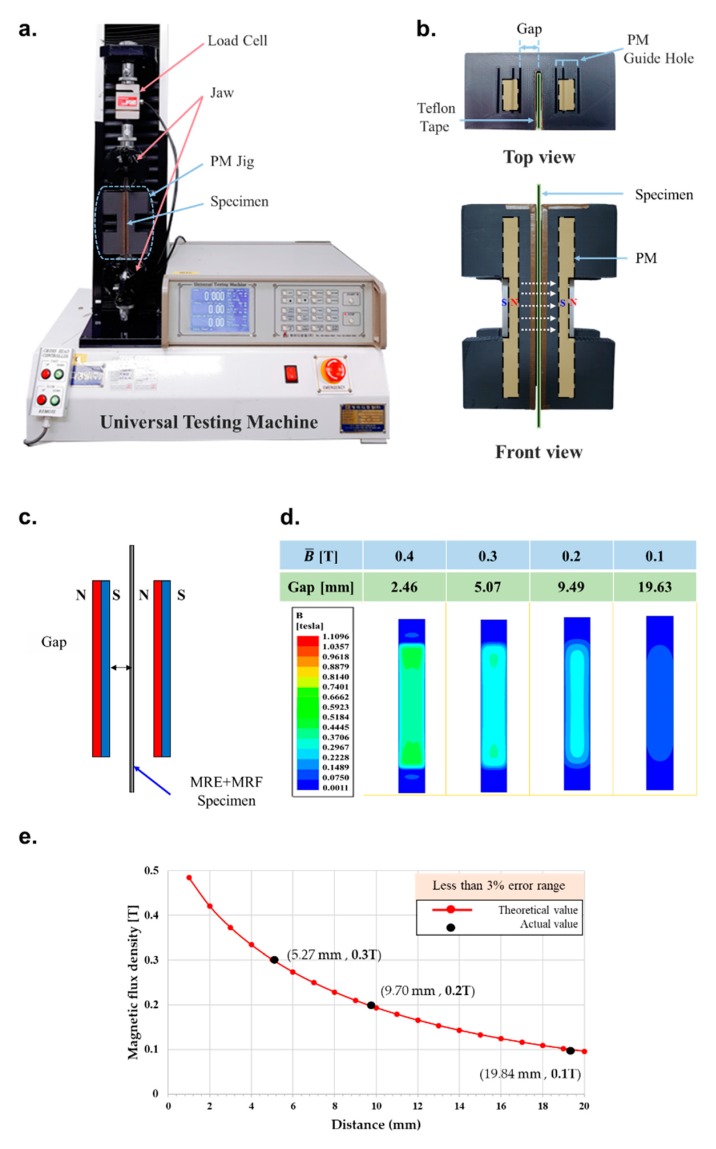
Experimental apparatus and set-up; (**a**) the universal tensile testing machine (KDPI-205 Series, KD PRECISION Co., capacity 1 kN), (**b**) top and front view of permanent magnet (PM) jig (**c**) the schematic diagram for the measuring distance and magnetic flux density, (**d**) the mean magnetic flux density simulation of the PMs based on FEM analysis, (**e**) the reference measurement of magnetic flux density using a gauss meter (F.W. BELL Co., 5100 series).

The change in stiffness of the soft structure as a function of the magnetic field intensity was investigated using the experimental apparatus and set-up shown in Figure 8. A universal testing machine (KDPI-205 Series, capacity: 1 kN, KD PRECISION Co., Seoul, Korea) was used as shown in Figure 8a, where the tensile force was detected by a load cell at the top and tensile length was determined by an embedded encoder attached to the motor. These two parameters were recorded by a data acquisition (DAQ) system to provide tensile length-tension in real time. The tensile speed was fixed at 500 mm/min and effectively tensed to 15 mm, except in the tow region where data was not collected due to the acceleration of the tensor before 500 mm/min was reached. The effects of friction on stiffness were minimized by attaching Teflon tape (coefficient of friction = 0.2–0.5) between the jig and the specimen as shown in Figure 8b. Two permanent magnets (PMs) with the same width as the soft structure were installed in a dedicated PM jig and used to apply the magnetic field in a specific direction across a large area. The PM jig included a section through which the specimen traveled upward and downward along the central axis. The magnetic field distribution with two PMs was measured using the finite element method (FEM) based on the gap distance shown in Figure 8c. The magnetic field distribution of the samples with PMs indicated that the PMs tend to change their magnetic flux density with increasing distance as shown in Figure 8d. Finite element analysis using commercial software (ANSYS Maxwell, ANSYS, Inc., Canonsburg, PA, USA) revealed that the average magnetic flux density ($\bar{B}$) was equivalent to 0.1 to 0.3 T. A plot comparing the FEM data with reference data measured using a gauss meter (F.W. BELL 5100 series, MEGGITT, Dorset, UK) indicated high accuracy and low errors of <3% (refer to Figure 8e).

## 4. Results and Discussion

**Figure 9:**
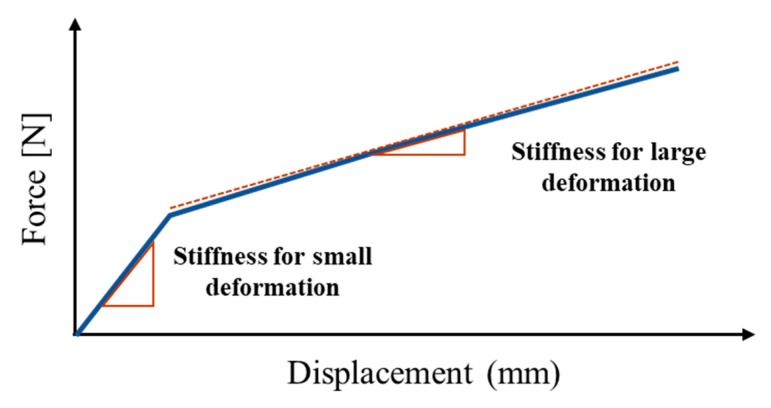
A typical linear approximated force-displacement curve of an MRE.

**Figure 10:**
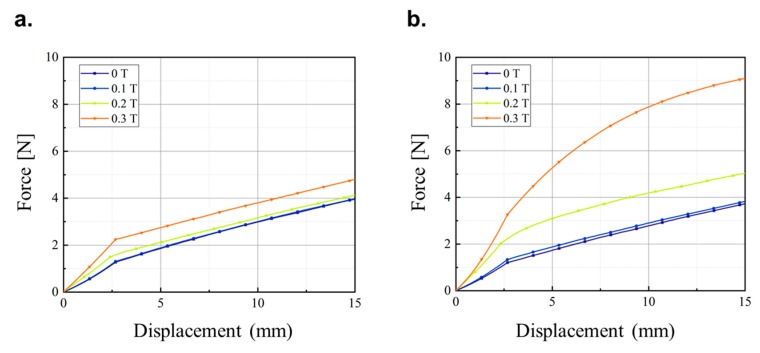
The tensile force-displacement curves in a range of magnetic flux density for the soft structures (**a**) without MRF and (**b**) with MRF.

The stiffness of the magnetic-responsive soft structures was compared to the mechanical properties of MRE structures in previous studies. The stiffness was defined as the slope of the force-displacement curve, but elastomers generally do not exhibit linear elastic deformation. Borin et al. [20] performed an elongation test on elastomers with magnetically hard powder and found that the modulus can be divided into two major ranges of deformation as shown in Figure 9. The stiffness behavior of the structure in this study is expected to be similar to that in the previous work as its main component, the skin layer, was an elastomer. The force-displacement curve of the soft structure across a range of magnetic flux density was measured (refer to Figure 10). The stiffness characteristics of the sample without MRF shown in Figure 10a were very similar to those in the previous study shown in Figure 9. The stiffness characteristics indicated that the small and large deformation regions separated at a deformation of 2.5 mm and the stiffness within each region was constant. A stronger magnetic field led to increased inter-particle interaction between the CIPs in the elastomer. This caused stiffening of the sample, which required a higher tensile force to cause deformation. However, this stiffening was only noted in the small deformation section and was qualitatively related to the action of CIP component of MRE. The inter-particle interaction was affected by the magnetic field when the particles were closer, i.e., in the case of small deformation. However, the distance between the CIPs in the large deformation region was too large and the particle interactions required to cause a change in stiffness change did not occur.

**Figure 11:**
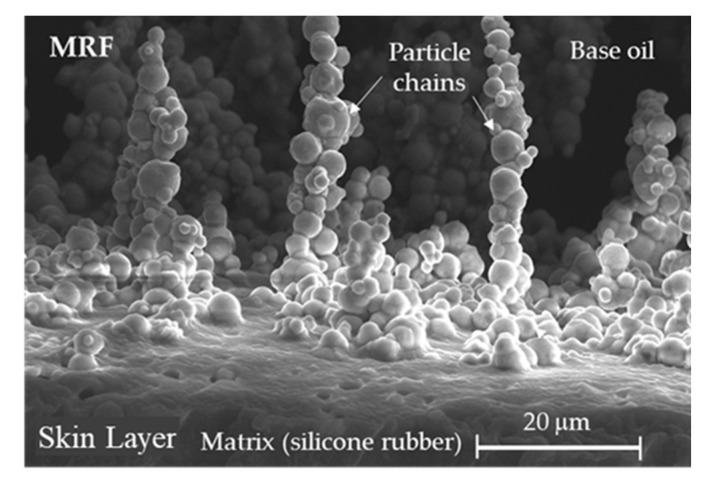
SEM image of the interface between the MRE skin layer and MRF core in the soft structure.

**Figure 12:**
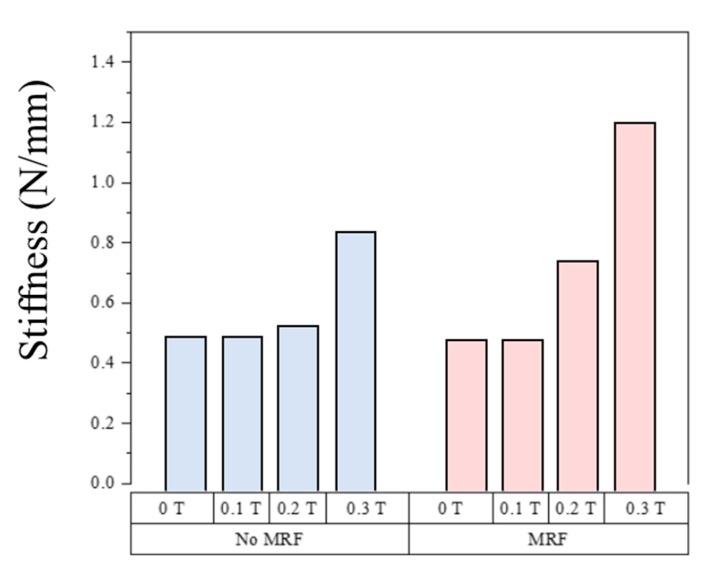
The stiffness was dependent on mean magnetic flux density for small displacement in the soft structures without MRF (blue) and with MRF (red).

The sample with MRF behaved completely differently as shown in Figure 10b. The modulus also exhibited small and large deformation regions separated at 2.5 mm deformation. However, unlike the sample without MRF, the slope for large deformation was not constant and the stiffness increased with increasing magnetic flux density. The magnetic field affected the stiffness of the soft structure with MRF despite the large deformation due to the microscopic structure of the MRF (refer to Figure 11). The SEM image (model: S-4300SE, Hitachi High-Tech Co., Tokyo, Japan) of the interface between the MRE skin layer and MRF core revealed that the CIP in the MRE and the CIP in the MRF interacted with one another because the CIPs in the liquid MRF can move more freely than the CIPs in the solid MRE skin layer. The stiffness changed according to the magnetic field even at large deformations, and the nonlinear relationship was attributed to the MR effect of the fluid in the presence of the magnetic field. For quantitative analysis, the stiffness in the small and large displacement regions across the magnetic field strength range was calculated for the samples with and without MRF. The change in stiffness according to the magnetic flux density in the small displacement region was based on an average slope because the tensile force increased linearly as shown in Figure 12. The stiffness of the sample without MRF increased by 72.8% when a 0.3 T magnetic field was applied, while the sample with MRF increased by 156.6% under the same conditions. The stiffness variation of the sample filled with MRF was greater than the sample without MRF due to the formation of chain-like structures in the MRF as shown in Figure 11.

**Figure A1:**
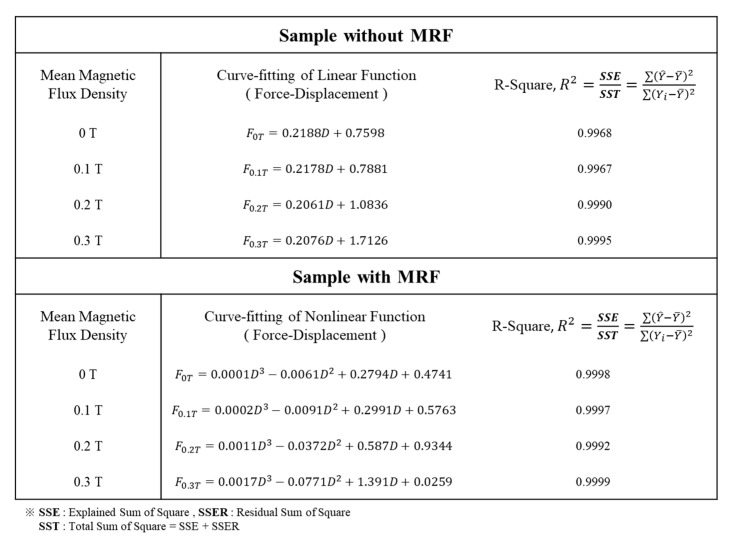
Force-displacement relationships approximated by polynomial expression in the large deformation region of the soft structure.

**Figure A2:**
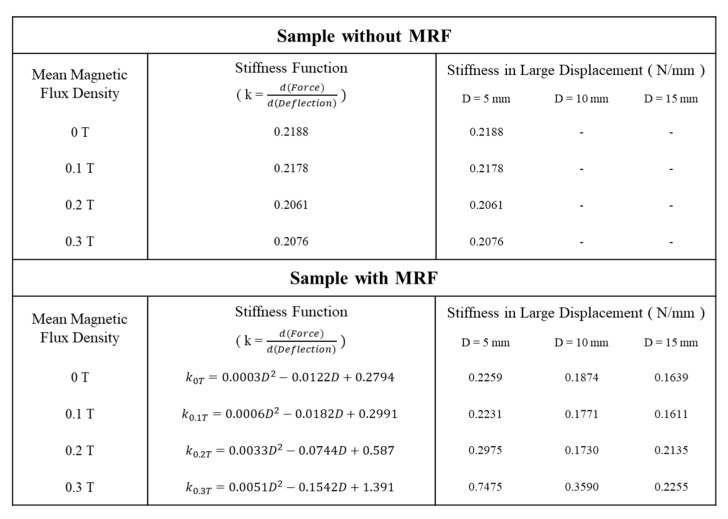
Stiffness approximated by polynomial expression in the large deformation region of the soft structure.

Unlike the small deformation region, stiffness did not increase linearly with displacement in the large deformation region and was instead characterized by an instantaneous slope based on the relationship between the tensile force and the displacement. Due to its nonlinearity, the tensile force-displacement curve was represented by a polynomial curve fitted as follows:

$$F\left(d\right)=\;\overset{n}{\underset{k=1}{\sum }}a_{k}*{\left(d-d_{b}\right)}^{k}$$

where *d*, *d_b_* and n denote the distance, bifurcation within large and small deformation and the order of polynomial expression, respectively. *F(d)* was fitted to order 3 and 1 for the samples with and without MRF, respectively. The order of the samples was chosen based on the tensile force-displacement curves given in Figure 10. Specific formulae and R-square values in the magnetic field intensity range were obtained and given in Figure A1 of Appendix A and the field-dependent stiffness for the large deformation region in Figure A2 of Appendix A. The stiffness was based on an instantaneous sloped force-displacement curve calculated by differentiating the values given in Figure A1. According to Figure A2, the stiffness of the sample without MRF did not change with displacement and was not affected by the magnetic flux density in the large displacement region. On the other hand, the stiffness of the sample with MRF changed according to displacement and was affected by the magnetic flux density. Notably, a 0.3 T magnetic flux density caused a 330.9% increase in stiffness at 5.0 mm deformation. The stiffness of the samples with MRF was higher than in samples without regardless of the amount of deformation, and a change in stiffness was noted even across a large deformation. The stiffness behavior of the soft structure proved that the use of a magnetic-responsive skin layer and core was very effective in controlling stiffness across a wide displacement or deformation range.

## 5. Conceptual Applications

**Figure 13:**
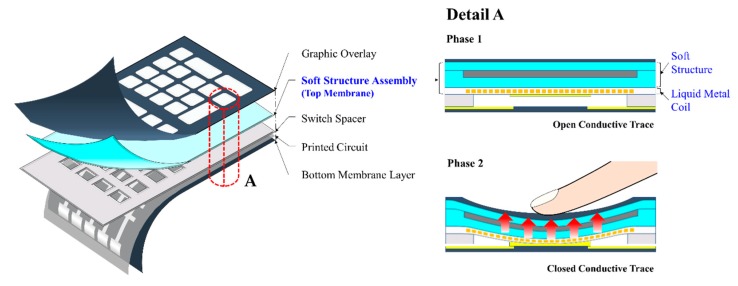
Schematic diagrams of a haptic rollable keyboard and its operating principles.

The new soft structure produced using two magnetic-responsive materials, namely MRE and MRF, with a stiffness tuned by external stimuli has been validated experimentally. Two conceptual applications utilizing the proposed soft structure were presented in this section in order to attract further interest in the present work for a wide range of applications. The first conceptual application is a rollable keyboard with a haptic function. There are two major issues associated with current keyboards, namely hardboards, which prevent portability, and the constant pressure of each key, which causes typos due to the lack of distinguishable pressure on each key. Both problems can be resolved by using the keyboard shown in Figure 13. By switching off the magnetic field, the keyboard loses its stiffness and can be easily rolled up and carried. Furthermore, the feedback force can be quantitatively adjusted and the haptic feedback of each channel can be controlled independently to achieve a different stiffness for each key. This will prevent typos in real time due to the different stiffness of each key. The haptic keyboard consists of a graphic overlay to provide visual information, a top membrane to act as a switch slider, a switch spacer to provide a gap between the top membrane and the circuit, a printed circuit to receive signals and a bottom membrane layer. The top membrane is a key component in achieving a soft structure, and a liquid metal coil produces the magnetic field when an input current is applied. As shown in Figure 13, Phase 1 represents the non-use state where the conductive trace is opened by the switch spacer and no current is applied to the liquid metal coil. Thus, no magnetic field is formed and the stiffness of the soft structure is very low. Phase 2 represents a state in which a key is pressed and an input current is applied to the liquid metal coil to form the magnetic field. The stiffness increases when pressed by the user and the haptic feedback causes the conductive trace to connect and produce a signal.

**Figure 14:**
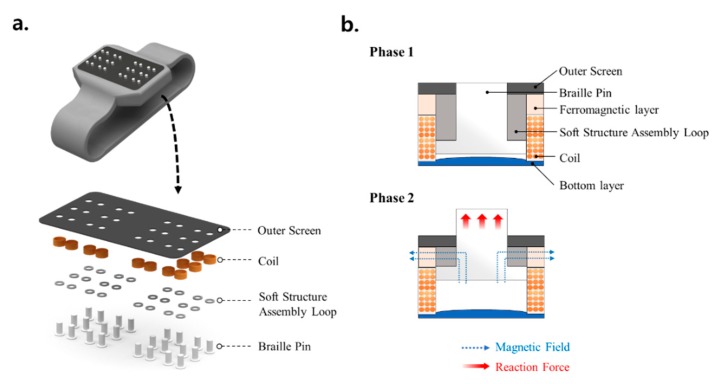
Schematic diagrams of a smart braille watch, specifically its (**a**) braille display actuator components and (**b**) operating principle.

The second conceptual example shown in Figure 14a is a wearable smart watch that can display braille for the visually impaired. Each braille pin has two states depending on its protruding height. If no current is applied to the coil, as shown in Phase 1 of Figure 14b, no magnetic field is applied to the soft structure assembly loop and the pin is positioned at a very low height. This is because the stiffness is low, and the braille pin is easily pushed downward when a user’s fingertips touch the outer screen. This causes tension between the soft loop connected to the bottom of the pin and the outer screen. The small deflection is not affected by the magnetic field and the reaction force is low. When the input current is applied to the coil, as shown in Phase 2, a magnetic field is formed around the circumference of the ferromagnetic layer, which is perpendicular to the tensile direction. The soft loop combined with MRF and MRE causes a large increase in stiffness when acted upon by the magnetic field. Therefore, there is a large tensile resistance at a small displacement that holds the braille pin at a high position. The misunderstanding caused by finger sensitivity and fatigue is prevented by adjusting the sensitivity of the braille pin attached to the soft structure.

## 6. Conclusions

A new soft structure with a stiffness tuned by external magnetic stimuli was produced, and the stiffness tuning capability of the structure was experimentally validated. The soft structure used two magnetic-responsive materials, namely an MRE as a skin layer and an MRF as a core. The MRE skin layer contained 30 wt.% carbonyl iron particles (CIP) and was manufactured through a multi-step process, including molding and curing. The rectangular mold with void channels to hold MRF was made from machine structure carbon steel (S55C). After bonding the upper and lower skin layers, two samples were produced, namely one with MRF-filled channels and one without (empty channels). Finite element analysis was conducted to investigate the magnetic field distribution with permanent magnets and the samples were fixed to the specialized jig in a universal tensile machine. The force-displacement curves of the samples with and without MRF were measured at a fixed tensile speed of 500 mm/min. The stiffness of the sample without MRF increased by 72.8% in the small deformation region when a 0.3 T magnetic field was applied, while the stiffness of the sample with MRF increased by 156.6%. The stiffness change in the large deformation region was also evaluated through curve fitting. The stiffness of the sample without MRF did not change in a magnetic field, while the stiffness of the sample with MRF increased by 330.9% in a 0.3 T magnetic field. The soft structure exhibited a stiffness change across a wide deformation range. The soft structure was proposed for use in two conceptual applications, namely a rollable portable keyboard and a smart wearable braille watch. These applications clearly demonstrate the great potential of this soft structure and should attract more interest in further applications of various fields including bioengineering.

However, in order to realize commercial products utilizing the proposed soft structures, several works need to be explored in depth. Firstly, analytical equation of motion and the finite element analysis of the proposed soft structure should be carried out to optimally fabricate any shape of the soft structure. It is remarked here that the finite element analysis of composite tubes characterized by different Young moduli has been investigated in [21]. Secondly, the permanent magnets to generate the magnetic field should be replaced by the magnetic core circuit to control each channel independently. This is crucial to get high magnetic flux density and control the stiffness of each channel to achieve the desired performance of application product. Thirdly, the field-dependent stiffness change should be identified in several directions including axial and bending motions. Furthermore, in order to increase the stiffness in the specific direction, the isotropic MRE used in this work should be replaced by the anisotropic by applying the magnetic field during the cure process. It is finally remarked that this work itself provides a proof-of-concept of the proposed soft structure whose tensile stiffness can be changed and hence controlled by the magnetic flux intensity. Therefore, the results presented in this work are preliminary which can attract a new research direction of the soft structures whose properties such as Young’s modulus can be instantly controlled by external stimuli.
